# A Functional MRI-Based Model for Individual Memory Assessment in Patients Eligible for Anterior Temporal Lobe Resection

**DOI:** 10.2174/1874440001711010001

**Published:** 2017-03-31

**Authors:** Maria Strandberg, Peter Mannfolk, Lars Stenberg, Hanna Ljung, Ia Rorsman, Elna-Marie Larsson, Danielle van Westen, Kristina Källén

**Affiliations:** 1 Department of Neurology and Clinical Sciences, Lund University Hospital, SE-221 85 Lund, Sweden; 2 Diagnostic Radiology, Department of Clinical Sciences, Lund University Hospital, SE-221 85 Lund, Sweden; 3 Department of Radiology, Uppsala University Hospital, SE-75185, Uppsala, Sweden

**Keywords:** FMRI, TLE, Verbal encoding, Lateralization index, Risk assessment, Verbal memory deficits

## Abstract

**Title:**

A functional (f) MRI-based model for individual memory assessment in patients eligible for temporal lobe resection.

**Aim:**

To investigate if pre-operative fMRI memory paradigms, add predictive information with regard to post-surgical memory deficits.

**Methods:**

Fourteen pharmacoresistant Temporal Lobe Epilepsy (TLE) patients accepted for Anterior Temporal Lobe Resection (ATLR) were included. A clinical risk assessment score (RAS 0-3) was constructed from structural MRI, neuropsychological testing and hemisphere dominance. fMRI lateralization indices (LIs) over frontal language and medial temporal regions were calculated. Predictive value from clinical risk scoring and added value from fMRI LIs were correlated to post-surgical memory change scores (significant decline -1 SD). Verbal memory outcome was classified either as expected (RAS 2-3 and post-operative decline; RAS 0-1 and intact post-operative verbal memory) or as unexpected (RAS 2-3 and intact post-operative verbal memory post-surgery; RAS 0-1 and post-operative decline).

**Results:**

RAS for verbal memory decline exhibited a specificity of 67% and a sensitivity of 75%. Significant correlations were found between frontal language LIs and post-operative verbal memory (r = -0.802; p = 0.017) for left (L) TLE and between medial temporal lobe LIs and visuospatial memory (r = 0.829; p = 0.021), as well as verbal memory (r = 0.714; p = 0.055) for right (R) TLE. Ten patients had expected outcome and four patients had an unexpected outcome. In two MRI-negative RTLE patients that suffered significant verbal memory decline post-operatively, fMRI identified bilateral language and right lateralized medial temporal verbal encoding. In two LTLE patients with MRI pathology and verbal memory dysfunction, neither RAS nor fMRI identified the risk for aggravated verbal memory decline following ATLR.

**Conclusion:**

fMRI visualization of temporal-frontal network activation may add value to the pre-surgical work-up in epilepsy patients eligible for ATLR. Frontal language patterns are important for prediction in both L and RTLE. Strong left lateralized language in LTLE, as well as bilateral language combined with right lateralized encoding in RTLE, seems to indicate an increased risk for post-operative verbal memory decline.

## 1 INTRODUCTION

Up to 30% of patients with temporal lobe epilepsy continue to suffer seizures despite advanced pharmacological treatment [1]. Surgery is a therapeutic alternative, specifically resection of the Anterior Temporal Lobe (ATLR), which is preceded by an extensive evaluation, as the procedure is not without risk. The Medial temporal lobe (MTL) structures are essential for memory and partial resection can cause cognitive deficits. Verbal and visuospatial memory decline are common deficits following temporal lobe epilepsy surgery [2].

Current methods for assessing the risk of post-operative memory decline include structural MRI, neuropsychological testing and language dominance assessment. The Intracarotid Amytal Test (IAT) for language lateralisation has in recent years been replaced by functional magnetic resonance imaging (fMRI), an advanced neuroimaging method relating to task-associated neuronal activation. Several studies point to a predictive value for assessment of post-surgical memory outcome [3-7]. Lateralized fMRI activation presented as lateralization indices, LIs, in relevant language areas have been shown to be the strongest predictor for verbal memory outcome following temporal lobe resection [6]. Most studies, however, present group data results, as the search for a predictive task at an individual level has not been without adversity.

The quest for a quantitative, prognostic memory task at an individual level continues and recent data point to the importance of testing both memory and language as they are closely linked [6, 8-10]. In patients with pharmacoresistant temporal lobe epilepsy material-specific re-organization can occur. Verbal skills are highly prioritized functions as left hemisphere dominant patients with left TLE often show a partial shift of verbal encoding to the right mesial temporal lobe structures, while verbal encoding in right, non-dominant hemisphere TLE patients with frequent seizures seems to remain unaffected and lateralized to the left temporal lobe [11].

Recent studies have increasingly used multivariate models and calculation of change scores to optimize predictions for memory decline post-ATLR. Such clinical parameters are neuropsychological test results, structural imaging findings, laterality of surgery, age of onset, disease duration, IQ and hemisphere dominance [7, 12]. These quantitative predictions provide a much more realistic picture of the actual outcomes, which are not dichotomous, but vary along a continuum.

The overall aim of this study was to explore the benefit from our previously published fMRI memory paradigm in patients under investigation for ATLR, with particular emphasis on individual assessment. The paradigm included an incidental verbal encoding task and a visuospatial memory task. LIs were calculated both for anterior language areas and for relevant structures in the medial temporal lobe. We hypothesized that verbal encoding in the MTL would activate pre-dominantly contralateral to the epileptogenic focus reflecting functional reorganization. Although not equally material-specific the visuospatial paradigm was included with the same notion. According to our hypothesis any deviation from this expected pattern would signal network disruption rather than reflecting a compensatory mechanism. The specific aim of this study was then to investigate the added value of the collected fMRI LIs to established clinical predictive factors known to have an impact on post-surgical verbal memory function.

## 2 METHODS

### 2.1 Subjects

Twenty-four adult Swedish-speaking patients with normal IQ and pharmacoresistant Temporal Lobe Epilepsy (TLE), both mesial and lateral, were included between November 2007 and August 2012. The Lund University Ethics Committee approved the study and all subjects gave their written informed consent. All patients were recruited from tertiary epilepsy surgery programmes in Sweden, mainly from the Skåne university hospital in Lund, but also from the university hospitals in (4) Göteborg and (1) Uppsala.

All patients had undergone neurological examination, structural MRI, pre-operative neuropsychological testing and extra-cranial video-EEG-recordings. Three patients had additional intracranial video-EEG, including subdural and hippocampal deep electrodes. The decision to offer temporal lobe resection was made at a multidisciplinary management conference and patients were included in the study after they were considered eligible for surgery.

Fourteen out of the twenty-four patients who performed the fMRI tasks before proceeding towards surgery were included in the analysis group: six patients had right and eight patients had left temporal lobe resections. Reasons for exclusion were not proceeding to operation (4), technical failure during scanning (3), lack of behavioural data supporting adequate task performance (2) and unrelated health issues (1).

The patients in the analysis group underwent ATLR with the aim of removing the anterior 3-4 cm of the temporal lobe, including lateral (middle temporal gyrus, inferior temporal gyrus, polar superior temporal gyrus) and medial (fusiform gyrus, parahippocampus, amygdala and anterior hippocampus) structures. Experienced neuropathologists in the regional epilepsy surgery teams evaluated the surgically removed specimens. Presurgical clinical data for each patient comprising sex, disease duration, age at disease onset, AED treatment, MRI lesions, hemisphere dominance assessed with fMRI and epileptogenic temporal lobe is presented in (Table **1**).

The classification of clinical seizure outcome was previously described by Edelvik *et al.* [13]. In short: seizure freedom (with or without aura); 75% reduction in seizure frequency; 50-74% reduction in seizure frequency; 0%-49% reduction in seizure frequency; and increased seizure frequency. Clinical follow-up time was median 54 months with a range of 5-80 months.

## 3 NEUROPSYCHOLOGICAL MEASURES

### 3.1 Pre-Operative Testing

A series of memory tests were drawn from the routine pre-surgical assessment protocol. Results were corrected for age and education. Parameters that reflect aspects of memory included encoding and retention. The Claeson-Dahl (CD) Learning and Retention Test [14] was used for examining verbal episodic memory. The CD list learning test enables assessment of encoding and retention through a word list with ten words which are orally presented and to be recalled after a delay of 15 seconds, over ten trials or until the entire list is correctly recalled twice. Retrieval of words (CD delayed recall) was assessed after 30 minutes.

The Rey Complex Figure Test (RCFT) [15] was applied for testing retention capacity of visuospatial material. The patients were asked to copy a complex figure, unaware that the same figure was to be recalled five and thirty minutes after the copying task. The latter measurement was used to reflect non-verbal/visuospatial retention.

We obtained three clinical neuropsychological variables:

- CD List learning (encoding)
- CD Delayed recall (retention)
- Rey Complex Figure Test delayed retention

Pre-surgical test results for each patient are presented in (Table **1**).

### 3.2 Post-Operative Testing and Assessment

Each subject was re-tested with the same psychometric battery post-surgery (median 6 months, range 3-20 months). CD list learning was considered to have a strong predictive value for post-surgical deterioration due to the fact that this parameter reflects the ability to establish new memories [16, 17]. A relative change, between pre- and post-surgical CD list learning scores, equal to or larger than 1.0 SD, was judged as significant in consistency with other studies of psychometric change [18, 19]. A change equal to, or larger than, 0.5 Standard Deviations (SD) was judged as a minor improvement or a minor deterioration (*i.e.* changes ranging from 0.5 to 0.9 SD). Minor deterioration in CD list learning in combination with a significant deterioration in CD delayed recall was also considered as a significant decline in verbal memory.

## 4 STRUCTURAL MRI

### 4.1 Pre-Operative MRI

Structural MRI scans were performed, as a part of the clinical investigation, at a 3T scanner following a protocol particularly designed for epilepsy surgery candidates including high resolution anatomical T1-weighted 3D magnetization prepared rapid acquisition gradient echo (MPRAGE) and T2-weighted fluid attenuated inversion recovery (FLAIR) sequences. All scans were reviewed by experienced neuroradiologists. Follow-up MRI scans were performed after surgery (median 6 months, range 2-15.5 months).

### 4.2 Post-Operative MRI

To measure resected volume and remaining hippocampus volume the cavity and the remaining hippocampus on the resected side were manually outlined on coronal MR-images reconstructed from 3D T1-sequences by an experienced neuroradiologist. Volumes were calculated by adding the areas of the regions of interest on each section and multiplying the total area by the slice thickness (1 mm). The anatomical landmarks were identified according to previously published anatomical MR-correlations [20] and are equal to the ones clinically used at our department for hippocampus volumetric measurements. The posterior limit of the hippocampal tail was defined as the section where the entire length of the crus fornices was seen. This section was not included in the volumetric determination.

## 5 FUNCTIONAL MRI

### 5.1 fMRI-Tasks - Experimental Design

The entire fMRI test comprised two different tasks: **(5.1.1.)** a verbal encoding paradigm and **(5.1.2.)** a visuospatial recall paradigm.

#### 5.1.1 The Verbal Memory Paradigm

The verbal fMRI task used to study memory effects in healthy subjects is described in detail in previous publications [21, 22]. Two different encoding tasks were mixed, one for deep and one for shallow encoding. 192 nouns were presented during scanning and in each noun two letters were underlined. Subjects were asked to perform one of two tasks: decide if the word was pleasant or not (deep encoding) or if the underlined letters were in alphabetical order or not (shallow encoding). Total scan time for the verbal task was 23:42 minutes.

#### 5.1.2 The Visuospatial Recall Paradigm

The second paradigm was a mental navigation task originally designed for a PET study, Roland Hometown Walking Task [23], later modified for fMRI use. The task is prepared before scanning where patients are asked to write down a familiar walk in their hometown, a walk divided into different stages. During scanning, the verbal description of each stage is projected on the screen and the patient is instructed to, in their mind, recall as many familiar details and landmarks as possible. The baseline task was to count odd numbers starting from 21. The task has been frequently used in fMRI studies [22-25] and it has demonstrated a predictive value for post-operative memory outcome [4]. Total scan time for the visuospatial task was 8:12 minutes.

An unexpected recognition test followed the scanning as we aimed at testing for incidental learning. Neuropsychological testing of incidental verbal encoding has shown to have a high ecological validity, *i.e.* correlate significantly to memory in everyday life [26]. On a computer screen the patients were consecutively presented with a random mix of the 192 nouns previously seen during scanning and an additional 96 words not seen before. They were asked to answer the following question: “have you seen this word during the scanning?” and answers were logged as subjects replied yes or no by pressing indicated mouse pad buttons. Logged answers were categorized and controlled as previously described [22]. The results served to ensure task compliance for the verbal encoding task. Patients who did not respond during the scanning or during the recognition task were excluded from the study.

### 5.2 fMRI Scanning

Functional magnetic resonance imaging was performed using a 3T Philips Achieva MR unit with an 8-channel head coil. A GRE-EPI pulse sequence (matrix size 64×64, TE = 30 ms, TR = 3000 ms, FoV = 192 mm, 49 slices, slice thickness = 3 mm, 0 mm slice gap, interleaved slice acquisition) was used for functional imaging. 3D T1-weighted and FLAIR (T2-weighted) sequences were used to obtain anatomical images for anatomical overlay of functional activation maps and to exclude pathology.

### 5.3 Preprocessing and BOLD Data Statistical Analysis

All data analysis was performed using MATLAB (Mathworks, Natick, MA). Preprocessing and statistical analysis was performed with SPM5 software (Wellcome Department of Cognitive Neurology, http://www.fil.ion.ucl.ac.uk/spm). Preprocessing included motion correction, where images were realigned to the first image to correct for movement-related variance, as well as slice time correction. For normalization the SPM5 EPI template was used, which is based on a standard Montreal Neurological Institute (MNI) space [27, 28]. Finally, the images were then spatially smoothed with an 8-mm isotropic Gaussian kernel to fulfill the assumptions of Gaussian random field theory [29].

Using the general linear model, statistical maps were computed for the verbal memory paradigm. Onset vectors were created from the logged data of each participant’s recognition test corresponding to all the possible event types. For the visuospatial task a single onset vector (active state) was created from the onsets of the eight blocks of mental navigation. The BOLD time course was modeled by convolving the onset vectors with the SPM5 canonical hemodynamic response function (HRF).

The following contrasts were analyzed:

(A) For the verbal memory paradigm:

**A**. The comparison of the deep encoding trials (“pleasant or not pleasant?”) with the shallow encoding trials (“correct alphabetical order or not?”) irrespective of successful encoding.

(B) For the visuospatial paradigm;

**B**. The active state (mental navigation) was contrasted against the baseline state (counting odd numbers).

The resulting contrast images were entered into a second level random effects analysis. The statistical parametric maps were thresholded at p<0.001, uncorrected for multiple comparisons. The threshold was chosen to reduce the occurrence of false positives as used in clinical practice at our department, for fMRI language and motor tasks.

### 5.4 Laterality Assessment

Laterality indices (LIs) were calculated using a toolbox running within the SPM environment [30]. Two masks for analysis of activation within regions of interest (ROI) were used.

- The **Broca ROI** was identical to the ROI used in our department for clinical lateralization of language using a word generation task. It includes the inferior frontal gyrus, the main part of the middle frontal gyrus and the dorsolateral pre-frontal cortex. This includes the area of Broca and its homologue in the right hemisphere.
- The **MTL ROI** encompassed the mesial temporal lobe structures that are relevant with respect to memory functions; hippocampus, parahippocampus, entorhinal cortex, perirhinal cortex and amygdala. The ROI was drawn using a standard single subject T1-weighted MR-image (ch2-template) in the MRICroN software [31] by an experienced neuroradiologist.

The bootstrap algorithm included as an option in the toolbox was applied in order to calculate robust LIs at different statistical thresholds (t-values). The algorithm was applied to masked statistical maps (t-maps) according to the previously defined ROIs obtained for the deep versus shallow contrast (the verbal paradigm, **A**) and the active versus baseline contrast (the visuospatial paradigm, **B**).

An overall LI mean greater than 0.1 was classified as left lateralized and an overall LI mean less than -0.1 was classified as right lateralized memory or language function. An overall mean LI between 0.1-(-0.1) was classified as bilateral (no lateralization), in accordance with previously used limits [22, 32, 33].

## 6 PREDICTION OF POSTOPERATIVE OUTCOME

### 6.1 Risk Assessment Score (RAS)

A comprehensive risk assessment score for prediction of postoperative memory decline was created for each patient taking into account hemisphere dominance, MRI-pathology and baseline memory capacity. Patients with good pre-operative memory and language function are at higher risk for developing post-operative deficits, particularly if the dominant temporal lobe is resected [16, 34, 35].

RAS parameters:

- Hemisphere dominance was mainly based on handedness classified in accordance with the Edinburgh Handedness Questionnaire [36] strongly emphasizing the hand used for writing. In our study all but one patient (# 1) were dexterous for writing. During the course of the clinical investigation this patient and three other patients performed a standard word generation fMRI task for lateralization of language (see Table **1**). Dominant resection received 1 point and non-dominant 0.
- For structural MRI, any potential epileptogenic lesion in the MTL was included. MRI-positive (0 points) refers to patients with any structural pathology noted pre-operatively as opposed to MRI-negative, *i.e.* normal finding (1 point).
- Baseline memory capacity was assessed with the CD list learning score [14]. The limit for good (*i.e.* normal) baseline memory was set at -1 standard deviation (SD) below the mean. In consistency with previous research [18, 19] any score below the normal range was considered to reflect memory impairment. Good baseline memory received 1 point and poor baseline memory received 0 points.

Patients with 0-1 points were considered as having a **low risk** for post-operative memory decline and 2-3 points as having a **medium**-**high risk** for post-operative deficits in verbal memory.

***Expected outcome*** was defined as:

RAS 2-3 followed by post-operative verbal memory decline

RAS 0-1 followed by no postoperative memory decline

***Unexpected outcome*** was defined as:

RAS 2-3 followed by post-operative intact memory

RAS 0-1 followed by post-operative memory decline

#### 6.1.1 Additional Risk in Relation to FMRI LI Indices

FMRI LI scores were individually evaluated for additional risk for verbal memory deterioration. The task was previously tested on healthy subjects [22] which, together with previous studies [11, 37, 38], generated the assumption that the fMRI activation would display the following patterns:

- For patients with left TLE, fMRI activity for language should be left lateralized in language regions and right lateralized for verbal encoding in the MTL.
- For patients with right TLE, fMRI activity for both language and encoding should be left lateralized.

Deviation from the expected pattern served as a red alert.

The possible estimation variables were:

- (0) No added risk: expected LIs for language and verbal encoding.
- (+) Possible added risk: deviation of expected LI for verbal encoding in the MTL ROI.
- (++) Added risk of verbal memory deficit: deviation of expected LI for language in the Broca ROI.

The basis for this grading was previous studies pointing to the important connection between language and encoding areas [6, 9, 10], where language lateralization fMRI was shown to have an even stronger predictive value than encoding fMRI activity [6].

For the visuospatial task, we assumed that fMRI activity should lateralize to the contralateral side of the epileptogenic focus, *i.e.* to the right MTL for LTLE patients and to the left MTL for RTLE patients [39]. As the task pertains to visuospatial recollection, not verbal encoding, it was not included in the risk assessment for fMRI indicating added risk for verbal memory decline. Fig. (**1**) illustrates the predicted fMRI patterns for LTLE and RTLE including the pattern for the various fMRI estimation variables.

## 7 STATISTICAL ANALYSES

Resected volumes, remaining hippocampal volume, age at disease onset and disease duration were all tested against psychometric outcome parameters. The Spearman correlation coefficient was calculated to test for correlations between the fMRI LI indices and psychometric outcome measurements. To investigate if the pre-operative assessment factors could be used to predict post-operative verbal memory decline, we performed multiple univariate logistic regressions and calculated odds ratio for all included parameters. We first calculated for all patients and then separately for the RTLE and LTLE group.

## 8 RESULTS

### 8.1 Pre-Op fMRI Data

Preoperative assessment of fMRI LIs for: language lateralization (Broca ROI), verbal encoding (MTL ROI) and visuospatial memory (MTL ROI) are shown in Table **2**.

RTLE group: Three out of six patients exhibited the predicted fMRI LI pattern (left lateralized language and verbal encoding). Two patients 4 and 5) fulfilled criteria for bilateral language representation; both had right-lateralized verbal encoding. Pat #6 had right (# -lateralized language and bilateral verbal encoding. All patients had, as expected, visuospatial memory lateralized to the left medial temporal lobe structures.

LTLE group: Five out of eight patients demonstrated the predicted fMRI LI pattern (left lateralized activity for language and right-lateralized activity for verbal encoding). Patients # 12 and 13 displayed left-hemisphere activation in the Broca ROI, but bilateral activation for verbal encoding in the MTL ROI. Patient 14 showed a highly unusual pattern for a right-handed individual with all lateralization indices pointing to the right hemisphere.

Two patients (#13 and 14) showed right-lateralized activity for visuospatial memory. Five patients exhibited left-lateralization - that is in their epileptogenic temporal lobe - for visuospatial memory.

Table **2** also shows the patients that, due to the fMRI LI indices pattern, have a perceived added risk for memory decline after ATLR.

**(0):** Eight patients were judged to have no increased risk according to fMRI indices as they exhibited the predicted lateralization pattern without any indication of bilateral language.

**(+):** Two patients (#12, 13; both LTLE) were judged to have a low (+) added risk indicated by fMRI. Both of them exhibited bilateral verbal encoding activity in the MTL.

**(++):** Four patients (RTLE patients #4, 5 and 6 and LTLE #14) were judged to have a significant (**++**) added risk as indicated by fMRI. They all displayed bilateral or right-hemisphere LIs in the Broca ROI and right-hemisphere or bilateral LIs in the MTL ROI for verbal encoding.

Three out of the four MRI-negative patients exhibited fMRI LI patterns indicating possible (#13 with LTLE) or significant added risk (#4 and 5 with RTLE) for postsurgical verbal memory decline.

### 8.2 Clinical Data After ATLR

Seizure outcome, resected volume, remaining hippocampal volume and neuropathological data are shown in Table **3** as well as the inter-test interval for each patient. Table 3 also relays the difference between before and after surgery for each patient in CD list learning, CD delayed recall and RCFT delayed recall. In summary, 10 patients were seizure free at the closing of this study. The most common histopathological finding was hippocampal sclerosis (N=10). The median resected volume was 24 cm^3^ (range 8-47 cm^3^), which is comparable to previous reports [40]. The median remaining hippocampal volume was 0.2 cm^3^ (range 0-2.4 cm^3^). There were no significant differences for volume resection or remaining hippocampal volume between RTLE and LTLE patients or when the patients were divided according to seizure outcome (seizure free (N=10) or not seizure-free (N=4)). Neither resection volume, remaining hippocampal volume or disease duration showed significant correlation to psychometric outcome parameters.

### 8.3 Individual Clinical Risk Assessment (RAS) and Memory Outcome

Table **4** includes the patients’ risk assessment scores and significant decline in verbal memory is specified for each patient. Three patients (#3, 4 and 5) in the RTLE group suffered a significant decline in verbal memory. In the LTLE group, five patients (#8, 9, 10, 11 and 13) suffered significant decline in verbal memory. For visuospatial recollection (RCFT), one patient (#1) in the RTLE group and one patient (#14) in the LTLE group suffered significant decline.

For the whole group, RAS for verbal memory decline exhibited a specificity of 67% and a sensitivity of 75%, numbers comparable to previous studies [12].

Ten patients had expected outcome (RAS 2-3 with verbal memory decline post-surgery or RAS 0-1 without verbal memory decline). Four patients had an unexpected outcome: RAS 2 and post-operative intact verbal memory (#7 and 12 with RTLE) or RAS 0-1 and verbal memory decline after surgery (#8 and 10 with LTLE).

### 8.4 fMRI Correlated to Post-operative Neuropsychological Data

As a group the only significant correlation was found between fMRI visuospatial memory LI in the MTL ROI and RFCT change score (r=0.586; p=0.035), *i.e.* stronger left-lateralization in the MTL during the mental navigation task correlated with better outcome for the visuospatial task. Figs. (**2** and **3**) shows the correlations between fMRI verbal encoding LI:s and CD change score in the MTL and the Broca ROIs, respectively, for LTLE and RTLE patients separately.

For the LTLE group there were two significant correlations:

- Between fMRI verbal encoding LI in the Broca ROI and CD change score (r= -0.802; p=0.017), *i.e.* stronger left-lateralization in the anterior language region during verbal encoding correlated with worse outcome verbal encoding Fig. (**2**)
- Between fMRI visuospatial memory LI in the MTL ROI and RCTF change score (r=0.829; p=0.021), *i.e.* stronger left-lateralization in the MTL during the mental navigation task correlated with better outcome for the visuospatial task.

**Fig. (3):**
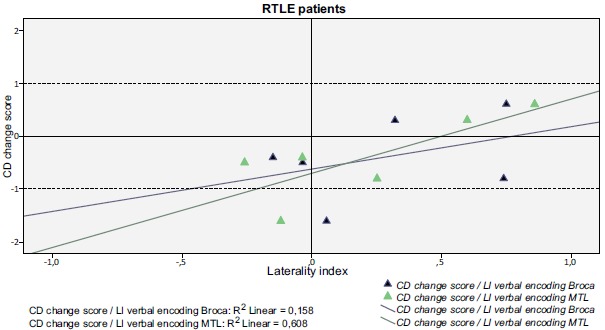
Laterality indices for verbal encoding in the Broca (coefficient of determination r^2^=0.158) and in the MTL ROI (coefficient of determination r^2^=0.608) respectively and their relation with CD change score are plotted for right TLE patients. A significant correlation between verbal encoding in the MTL and subsequent decline in verbal memory was seen (r=0.714; p=0.055).

For the RTLE group there was a marginally significant correlation between fMRI verbal encoding LI in the MTL ROI and CD change score (r=0.714; p=0.055), *i.e.* stronger left-lateralization correlated with better outcome in list learning (Fig. **3**).

None of the odds ratios were statistically significant.

### 8.5 Individual fMRI Risk Assessment and Post-op Verbal Memory Deficit

Pre-operative verbal encoding fMRI in the MTL and the Broca ROI identified the risk for postoperative memory decline in two patients (# 4 and 5). Both underwent right ATLR, pre-operative MRI was normal and RAS scored 2. fMRI indicated an added risk due to bilateral language representation combined with right lateralized verbal encoding in the MTL.

Pre-operative verbal encoding fMRI in the MTL only indicated increased risk (“red alert”) for two patients (# 12 and 13), but this risk was realized only for one patient (#12).

In neither of the two patients (# 8 and 10) that suffered an unexpected verbal memory decline post-operatively did fMRI indicate increased risk. Nor was the indicated risk realized for patient # 6 and 14.

## 9 DISCUSSION

Assessing and correctly predicting the risk for verbal memory decline is an important and still unresolved issue in epilepsy surgery investigations. In this study we investigated conventional clinical risk assessment together with fMRI laterality indices from a verbal encoding paradigm to improve prognostication of post-operative verbal memory decline at an individual level. We found that frontal language patterns are important for prediction in both left and right TLE. Strongly left lateralized language in LTLE, as well as bilateral language and right lateralized medial temporal lobe encoding in RTLE, seems to indicate an increased risk for post-operative verbal memory decline. The comprehensive fMRI paradigm produces versatile data for both verbal and visuospatial memory.

Increased language lateralization to the left in the Broca ROI during verbal encoding correlated with post-operative decline of verbal memory for LTLE patients. The more the fMRI activity in anterior language areas was left lateralized, the greater was the decline in verbal memory following surgery. This, in part, calls to mind the results of Binder [9] and may also lend support to the functional adequacy model which predicts that post-operative memory outcome will be inversely related to the level of preoperative functioning of the tissue to be resected [34, 41, 42].

The functional adequacy model has received sustenance from fMRI studies aiming at prediction. Focus has been on the to-be-resected tissue (the anterior medial temporal lobe), but the functional adequacy tissue of importance should include the collaborative network of the to-be-resected tissue as well, namely the language network of the anterior frontal lobe. This proposition becomes increasingly valid considering the growing interest for medial TLE as a network disease with widespread network disruptions, and the idea that intrinsic connectivity reflects behavioral capacities [43]. A call for attention to, and more extensive characterization of, language capacity in several domains in epilepsy patients has recently been made [44]. Recently, it has been shown that fMRI activations outside the temporal lobe may have a role in the prediction of verbal memory [45]. Our results support these propositions: three out of the four MRI-negative patients that suffered significant verbal memory decline could be identified by unexpected fMRI patterns, suggesting that functional network changes precede structural changes visualized by current structural MRI.

Regarding the fMRI indices for language and verbal encoding in the LTLE group most data confirmed the expected clinical risk assessment of memory decline. However, four left hemisphere dominant patients had unexpected verbal memory outcome. Two (# 8 and 10) of them had unexpected decline and two (#7 and 12) - although judged to be at high risk - did not show significant verbal memory decline by our definition. Traditional clinical parameters did not identify any of these cases. In one patient (#12) the fMRI index for verbal encoding raised a warning flag by indicating pre-operative equal use of both MTLs for verbal encoding, though this warning proved inaccurate.

One of our most interesting findings pertains to patients eligible for RTLR. For two out of three RTLE patients with a medium-high risk assessment score, bilateral fMRI language patterns combined with right lateralized verbal encoding in the medial temporal lobes structures raised additional alert. RTLE patients are not traditionally considered to be at great risk for post-operative verbal memory decline although it has been previously described [46, 47]. Studies have shown post-operative verbal memory deficits in 20-24% of non-dominant TLE patients [46, 48] and the prediction of risk patients in this group is probably a neglected field of interest [49]. Furthermore, a recent study established an equal risk for post-operative memory decline in both RTLE and LTLE patients with unilateral HS and intact verbal memory before surgery [50]. The authors concluded that their findings did not support that intact memory was a function of migration to the contralateral structures in people with unilateral HS. Our results are in agreement with theirs and emphasize the importance of the networks between language areas and the MTL structures; particularly considering the impact hippocampal pathology can have on language patterns [51]. Although language function is less investigated than memory in TLE, it has been shown that 17-33% of these patients have language deficits [44] associated with hippocampal sclerosis [52, 53].

In our study, three of the six patients eligible for non-dominant resections received an (++) added risk due to unanticipated fMRI LI indices. Two of them (#4 and 5) did suffer verbal memory decline post-surgery. In lieu of visible HS on MRI and poor pre-operative verbal memory, the fMRI indices for patients #4 and 5 added valuable information. Functional reorganization does take place in all patients and in terms of fMRI activation it is still unclear what “atypical activation” represents [54]. In this case, we interpret the LIs as an indication of atypical language representation coupled with an atypically organized verbal memory, increasing these patients’ vulnerability to intervention in non-dominant MTL structures. The fMRI pattern for patient # 6 is similar to that of patients 4 and 5, but she received a very low risk assessment score. Although her fMRI patterns indicated increased risk, her poor pre-operative memory probably decreased the absolute risk of further decline.

Correlation between language (LI Broca) and verbal encoding LI in the MTL for the RTLE group emphasizes the importance of visualizing connections between language and verbal encoding in the non-dominant hemisphere. The basis for the co-lateralization hypothesis is that the temporal lobe receives input from the ipsilateral cortex, thus creating the setting for verbal or nonverbal material specificity in the MTL. FMRI was recently suggested to be more sensitive to right-hemisphere processing than the IAT procedure [55], hopefully benefitting right TLE patients.

Out of the fourteen subjects, eleven showed left-lateralization for the visuospatial task making it one of the most consistent findings in our study. We did not include the visuospatial task in our pre-trial assumptions for several reasons, primarily because its material-specificity was not our main focus. Also, RCFT is not considered a strong predictor of non-dominant temporal lobe function [18]. Test results have not been able to differentiate between RTLE and LTLE groups [56], due to the fact that the task can be easily verbalized and performed with either visuospatial recollection or internal verbalization. The visuospatial task has previously been shown to reliably activate MTL structures - bilaterally and symmetrically - in healthy subjects [20, 36]. For TLE subjects, it has shown reduced activity ipsilateral to the epileptogenic side [4], but a corresponding psychometric task was lacking. We were only able to reproduce their findings at the group level. Two patients suffered significant decline in RCFT change score, one patent had RTLE with left lateralized visuospatial encoding (#1), one had LTLE with right lateralized visuospatial encoding (#14).

A limitation of our study was that a standard fMRI language lateralization was not generally performed in all patients eligible for TLR, a limitation also present at our clinical facility and at referring centers at the time of the trial. The decision, however, to study the Broca region and not a more encompassing range of language network, was based on the traditional approach to lateralize, not localize, anterior language regions using fMRI. Although atypical language lateralization is more common in epilepsy patients [57], hemisphere dominance was assumed based on handedness rating scale results, family history of left-handedness and pre-surgical domain-specific neuropsychological deficits. Patient #14 revealed unusual fMRI indices for a right-handed person: all indices pointed towards dominance in the right hemisphere. The patient was ambidextrous but dexterous for writing and eligible for “dominant” left ATLR. The patient improved in list learning (immediate, but not delayed recall) and worsened in visuospatial recall after LTLR, an uncharacteristic profile for a “dominant” resection. MRI and later histopathological examination verified HS, a finding that, as previously noted, is connected with atypical language patterns [50].

Our results should be regarded with caution. First and foremost the low number of subjects, the main limitation of our study, makes any result, even statistically significant correlations, preliminary at best. The number of participants also prevented us from using multivariate models, which produce notably better predictive information. The group heterogeneity, with even smaller subgroups, also prevented us from performing further activation pattern analysis, group analysis of t-maps or data-driven within-ROI analysis to look for activated regions with greater outcome correlations. The novelty of our study is that the comprehensive fMRI paradigm seems to provide predictive information for both verbal and visuospatial memory, on a group - however small - level.

## CONCLUSION

From our study, we conclude that fMRI indices seem to add value, although not above and beyond the RAS; to the pre-surgical work-up of epilepsy patients eligible for ATLR. Conventional clinical predictive markers can be improved by non-invasive functional MRI of cognitive function as they reflect different aspects of cognition, thus improving prediction of post-operative decline and patient counseling. All patients eligible for resection probably benefit from the determination of language lateralization. Language patterns are important for both left and right TLE patients, as memory and language co-lateralize and bilateral language or right hemisphere dominance is common. For left TLE patients left-lateralized fMRI activity correlated with post-operative verbal memory decline at the group level. Among the right TLE patients, two patients that suffered a post-operative verbal memory decline were identified by fMRI activity for language and verbal encoding, where bilateral language activation patterns seemed to indicate increased vulnerability. Our study emphasizes the need for further studies focusing on medial temporal lobe epilepsy as a network disease where the connections between the many functional areas and clinical correlates are explored.

## Figures and Tables

**Fig. (1):**
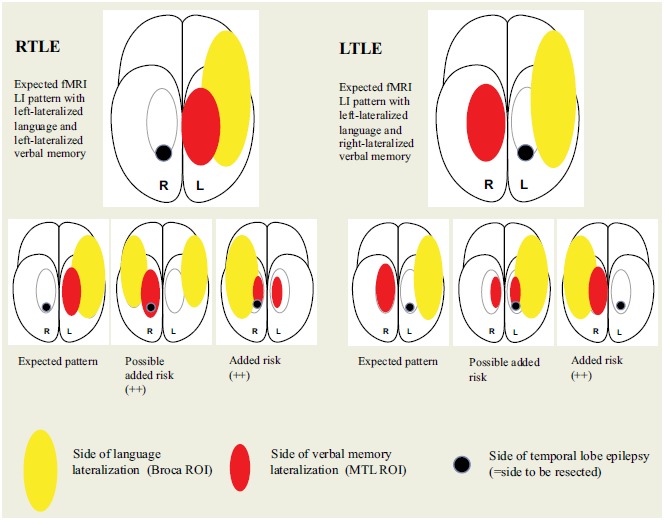
Illustration of the predicted fMRI patterns for LTLE and RTLE including the number of patients in the various fMRI estimation variables.

**Fig. (2):**
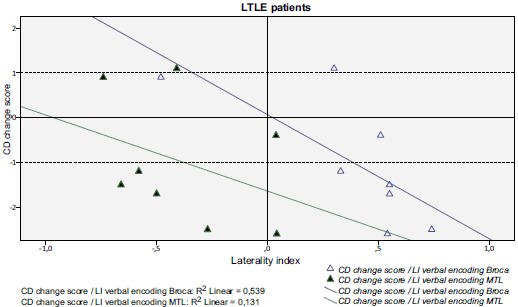
This figure shows the laterality indices for verbal encoding in the Broca (coefficient of determination r^2^=0.539) and in the MTL ROI (coefficient of determination r2^=^0.131). A significant, inverse correlation (r=-0.802, p=0.017) was seen for activity in the Broca ROI during verbal encoding and subsequent CD change score (decline in verbal memory) for left TLE patients.

**Table 1:** Pre-operative clinical data: sex, age at disease onset, disease duration, anti-epileptic drugs (AEDs), epileptogenic temporal lobe (defined by EEG seizure onset), MRI, hemisphere dominance assessed by fMRI and pre-operative neuropsychological test results.

Nr/Id/Sex	**Age at disease onset**	**Disease duration (yrs)**	**AED at time of investigation**	**Epileptogenic temp lobe (to be resected)**	**Hemisphere dominance by word generation fMRI**	**MRI pathology**	**CD List recall** **(SD)**	**CD Delayed recall** **(SD)**	**RCFT** **delayed recall ** **(SD)**
1/M	28	13	CBZ	R TL	Left	R mesial sclerosis	-0.5	0.3	-0.5
2/F	17	18	LTG, GBP	R TL	Not available	R occipital demarcated infarction; R hc atrophy	0.3	0.5	-2.2
3/M	47	7	LTG, VPA	R TL	Left	0	0.5	0.7	0.7
4/F	34	5	LEV, CBZ, PGB	R TL	Not available	0	1	0.6	-1.3
5/F	34	4	CBZ	R TL	Not available	0	-0.5	-0.2	-2.3
6/F	8	19	LTG	R TL	Not available	R mesial sclerosis	-1.6	-2.5	-2.6
7/M	15	32	LEV	L TL	Not available	L TL suspect DNET	-0.7	-1.3	0.5
8/M	19	4	VPA, LTG	L TL	Left	L TL suspect DNET	-1.4	0.1	-1.9
9/F	1	31	LTG, CLB	L TL	Not available	L mesial sclerosis	0.1	-2.1	-1.1
10/F	19	12	PGB, CBZ	L TL	Not available	L mesial sclerosis	-3.3	-2.7	--*
11/M	25	5	OXC	L TL	Not available	L TL low-grade astrocytoma	0.8	-4.5	-1.6
12/F	10	20	LTG, VPA	L TL	Not available	L mesial sclerosis	-0.2	-0.1	0.0
13/F	13	11	LTG	L TL	Left	0	-0.8	-0.8	-1.2
14/F	32	28	LEV, OXC	L TL	Not available	L mesial sclerosis	-2.2	-0.5	-1.3

*Not able to perform CBZ Carbamazepine; LTG Lamotrigine; GBP Gabapentin; VPA Valproic Acid; LEV Levetiracetam; PGB Pregabalin; CLB Clobazam; OXC Oxcarbazepine. L Left; R Right; TL Temporal Lobe; DNET Dysembryoplastic Neuroepithelial Tumour; SD Standard Deviation; CD Claseson-Dahl Learning and Retention Test; RCFT Rey Complex Figure Test

**Table 2:** fMRI laterality indices and added risk for verbal memory decline as indicated by fMRI LI pattern.

**Nr/ID**	**Verbal encoding LI** **Broca ROI**	**Verbal encoding LI MTL ROI**	**Visuospatial memory** **LI MTL ROI**	**Added risk indicated by fMRI indices**
1	0.75	0.86	0.15	0
2	0.32	0.6	0.62	0
3	0.75	0.25	0.14	0
4	0.056	-0.12	0.39	++
5	-0.036	-0.26	0.15	++
6	-0.15	-0.037	0.66	++
7	0.3	-0.41	0.4	0
8	0.33	-0.58	0.52	0
9	0.55	-0.66	0.33	0
10	0.55	-0.5	0.53	0
11	0.74	-0.27	0.003	0
12	0.51	0.039	0.22	+
13	0.54	0.042	-0.46	+
14*	-0.48	-0.74	-0.36	++

*Right-handed for writing, but ambidextrous for other activities

**Table 3:** Post-operative clinical data: seizure outcome, neuropsychological change score, inter-test interval for each patient, histological findings and resected volumes based on pre-and post-operative MRI comparison.

**Nr/Id**	**Seizure free**	**Neuropsychological change score (SD) post-op:** **CD list learning/CD delayed recall/RCTF delayed recall**	**Interval between pre- and post-operative assessment (months)**	**Neuropathology**	**Resected volume **(cm ^3^)	**Remaining hippocampal volume **(cm ^3^)
1	No (50-74% sz ↓)	0.6/0.1/-1.7	5.5	Hc sclerosis + neuronal heterotypes	28	*
2	No (75% sz ↓)	0.3/-0.5/0.6	18	Hc sclerosis	25	0.2
3	Yes	-0.8/-2.1/0	8	Hc sclerosis	39	0.2
4	Yes	-1.6/0/0.5	7.5	Hc sclerosis	24	0.3
5	Yes	-0.5/-1.1/-0.7	10	Discrete disturbance of neuronal migration	47	0
6	Yes	-0.4/0.8/-0.4	7	Hc sclerosis + gliosis	22	1.6
7	No (75% sz ↓)	1.1/-0.7/0.7	4	Gliosis	8	1.9
8	Yes	-1.2/-0.1/1.2	20	Neuronal heterotypes, glio-neurovascular dysplasia	25	0.1
9	Yes	-1.5/-0.9/0.3	12	Hc sclerosis	22	0.1
10	Yes	-1.7/-1.8/--	6.5	Hc sclerosis + neuronal heterotypes	19	0.2
11	Yes	-2.5/3.1/-0.4	24	Astrocytoma grade I-II	27	2.4
12	Yes	-0.4/0/1	12	Hc sclerosis	31	0
13	No (75% sz ↓)	-2.6/-0.5/-0.4	19	Hc sclerosis	18	0.5
14	Yes	0.9/-2.1/-1.7	11	Hc sclerosis	17	0.3

*Not possible to measure due to poor image quality. Sz seizures; SD standard deviation; hc hippocampal.

**Table 4:** Individual risk assessment score and significant verbal memory decline.

Nr/ID	Hemisphere dominance (**1=dominant, *i.e.* L (left); 0=non-dominant, *i.e.* R (right)**	MRI pathology **(1=no; 0=yes)**	Pre-op verbal memory (**list learning) score (Lower than -1SD (↓)=0; higher than -1SD (↑)=1)**	RAS - risk assessment score	Significant verbal memory decline
1	0 R	0 yes	1	1	No
2	0 R	0 yes	1	1	No
3	0 R	1 no	1	2	Yes
4	0 R	1 no	1	2	Yes
5	0 R	1 no	1	2	Yes
6	0 R	0 yes	0	0	No
7	1 L	0 yes	1	2	No
8	1 L	0 yes	0	1	Yes
9	1 L	0 yes	1	2	Yes
10	1 L	0 yes	0	1	Yes
11	1 L	0 yes	1	2	Yes
12	1 L	0 yes	1	2	No
13	1 L	1 no	1	3	Yes
14	1 L	0 yes	0	1	No
